# A nonlinear controlling function of geological features on magmatic–hydrothermal mineralization

**DOI:** 10.1038/srep27127

**Published:** 2016-06-03

**Authors:** Renguang Zuo

**Affiliations:** 1 State Key Laboratory of Geological Processes and Mineral Resources, China University of Geosciences, Wuhan 430074, China

## Abstract

This paper reports a nonlinear controlling function of geological features on magmatic–hydrothermal mineralization, and proposes an alternative method to measure the spatial relationships between geological features and mineral deposits using multifractal singularity theory. It was observed that the greater the proximity to geological controlling features, the greater the number of mineral deposits developed, indicating a nonlinear spatial relationship between these features and mineral deposits. This phenomenon can be quantified using the relationship between the numbers of mineral deposits *N*(*ε*) of a *D*-dimensional set and the scale of *ε.* The density of mineral deposits can be expressed as *ρ*(*ε*) = *Cε*^−(*D*e−*a*)^, where *ε* is the buffer width of geological controlling features, *D*e is Euclidean dimension of space (=2 in this case), *a* is singularity index, and *C* is a constant. The expression can be rewritten as *ρ* = *Cε*^*a*−2^. When *a* < 2, there is a significant spatial correlation between specific geological features and mineral deposits; lower *a* values indicate a more significant spatial correlation. This nonlinear relationship and the advantages of this method were illustrated using a case study from Fujian Province in China and a case study from Baguio district in Philippines.

The formation of magmatic–hydrothermal ore deposits is strongly controlled by geological features, such as intrusions and faults; these provide the heat sources and hydrothermal pathways required for magmatic–hydrothermal mineralization. In mineral exploration, the methods used to express and quantify the controlling function of these geological features on magmatic–hydrothermal mineralization are critical for understanding the formation of ore deposits and for assigning the weights to these features. However, these methods have not been extensively researched. The Student’s *t* = *c*/*S*(*c*) has been widely used to measure the spatial association between geological controlling features and mineral deposits using the weights of evidence (WofE) method^1^,^2^, which is a popular data-driven approach for mapping mineral prospectivity. Here, *c* is equal to *W*^+^−*W*^−^; *W*^+^ and *W*^−^ are positive and negative weights used when a geological controlling feature is present or absent, respectively; *S*(*c*) is the standard deviation of *c*. Calculation of these parameters is described by Bonham-Carter *et al*.^2^. A Student’s *t* value > 1.96 indicates that the geological controlling feature is spatially correlated with known mineral deposits at a 95% confidence interval. The higher the Student’s *t* value is, the stronger the spatial association is between a specific geological controlling feature and mineral deposits. However, this method does not reveal the geological controlling function on mineralization, and the calculated Student’s *t* value is influenced by the grid size selected. The aim of this paper is to report a nonlinear controlling function of geological features on magmatic–hydrothermal mineralization, and to describe an alternative method to measure the spatial association between geological features and mineral deposits using multifractal singularity theory.

## Results

### Skarn Fe deposits in Fujian Province, China

**Figure 1:**
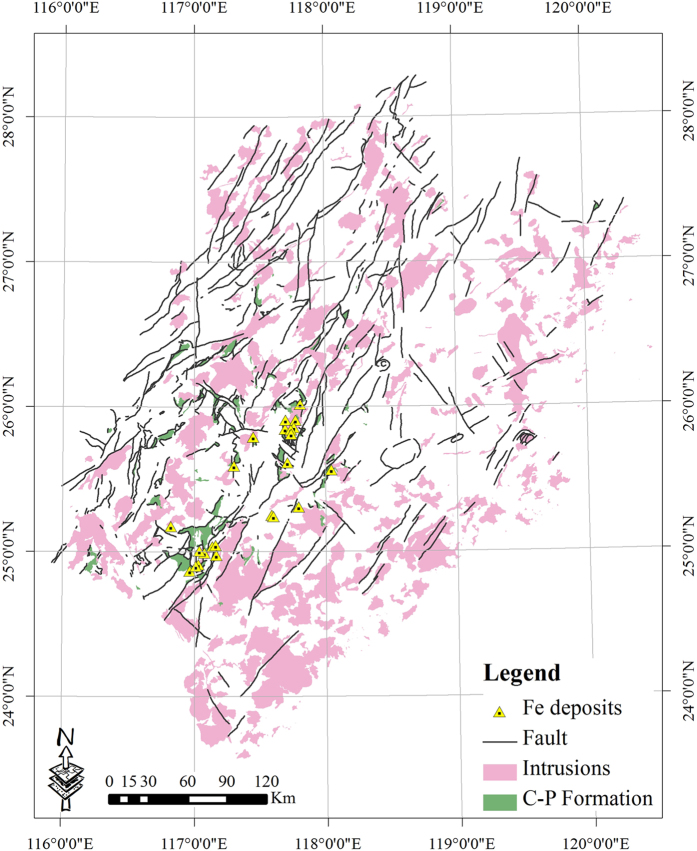
Simplified geological map of the Fujian metallogenic belt in China 3,4. This map was created using ArcGIS 10.2 (http://www.esri.com/software/arcgis/arcgis-for-desktop).

**Figure 2:**
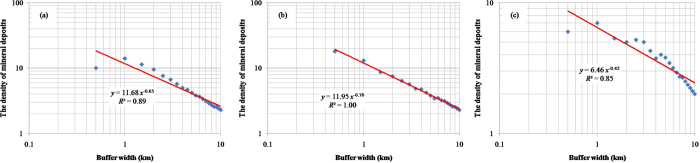
Log–log plots of density of mineral deposits versus buffer width for (**a**) Jurassic to Cretaceous (Yanshanian) intrusions, (**b**) Late Palaeozoic marine sedimentary rocks and carbonate formations (C–P Formation), and (**c**) NNE–NE-trending faults.

Zuo *et al*.^3^ and Wang *et al*.^4^ indicated that skarn Fe mineralization in Fujian Province (China) is controlled by three key geological factors: Jurassic to Cretaceous (Yanshanian) intrusions, Late Palaeozoic marine sedimentary rocks and carbonate formations (C–P Formation), and NNE–NE-trending faults (Fig. 1). The data used in this study, which pertained to intrusive rocks, formations, faults, and 23 Fe deposits were compiled from Zuo *et al*.^3^ and Wang *et al*.^4^. Within the Fujian Province, the Makeng is one of the largest Fe deposits with an ore reserve of more than 350 Mt and an average grade of 37.85% total Fe^5^. The singularity indices for the three above-mentioned geological factors, as obtained by the proposed method, were 1.35, 1.30, and 1.58 (Fig. 2), respectively, indicating that their controlling function on Fe mineralization is nonlinear. The spatial associations between Yanshanian intrusions/C–P Formation and mineral deposits were more significant than in the case of NNE–NE-trending faults. When comparing these singularity indices with the Student’s *t* values, it can be noted that the greater the Student’s *t* value (>1.96), the lower the singularity index *a* (<2) (Table 1), indicating that both approaches are effective for measuring the spatial relationship between geological features and mineral deposits. Based on the values of the singularity index and Student’s *t*, the order of importance of these controlling functions on skarn Fe mineralization is: C–P Formation >Yanshanian intrusions >NNE–NE-trending faults^3^,^4^.

### Epithemal Au mineralization in Baguio district, Philippines

**Figure 3:**
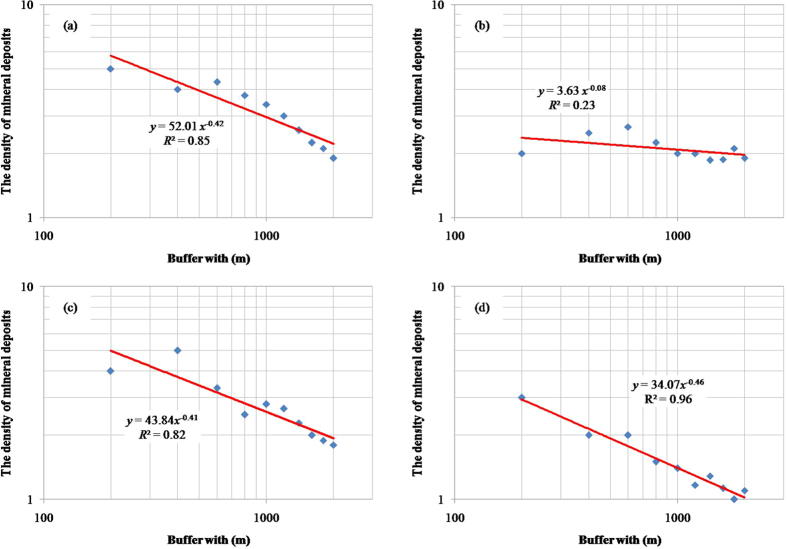
Log–log plots of density of mineral deposits versus buffer width for (**a**) NE-trending faults/fractures, (**b**) NW-trending faults/fractures, (**c**) Batholithic pluton margins, and (**d**) porphyry pluton contacts.

Carranza and Hale^6^ identified four geological features related to epithemal Au mineralization in Baguio district (Philippines): NE-trending faults/fractures, NW-trending faults/fractures, batholithic pluton margins, and porphyry pluton contacts. These four geological features and locations of 19 large-scale gold occurrences digitized from Carranza and Hale^6^ were extensively studied for mapping gold mineral prospectivity^6^,^7^,^8^,^9^. Log–log plots of the buffer width of these geological features versus the density of mineral deposits (Fig. 3) show that the gradients of the fitted lines were −0.42, −0.08, −0.41, and −0.46; the obtained values of the singularity indices for NE-trending faults/fractures, NW-trending faults/fractures, batholithic pluton margins, and porphyry pluton contacts were 1.58, 1.92, 1.59, and 1.54, respectively (Table 1). These results suggest that the controlling functions of NE-trending faults/fractures, batholithic pluton margins, and porphyry pluton contacts on Au mineralization are nonlinear, and these three geological features are spatially correlated with Au mineralization due to their singularity indices being <2. The singularity index for the batholithic pluton margins (1.54) is lower than that for NW-trending faults/fractures (1.58) and porphyry pluton contacts (1.59), indicating that their spatial controlling function is more significant than those of NW-trending faults/fractures and porphyry pluton contacts. The singularity index of NW-trending faults/fractures approaches 2, suggesting a weak spatial association between NW-trending faults/fractures and Au mineralization. The Student’s *t* values^8^ for these four geological features exhibit a similar trend (Table 1), being >1.96 for NE-trending faults/fractures, batholithic pluton margins and porphyry pluton contacts, and thus suggesting a strong spatial correlation with Au mineralization. However, the Student’s *t* value for NW-trending faults/fractures is <1.96, indicating a non-significant spatial relationship.

## Discussion

Analysis of magmatic–hydrothermal mineralization, in Fujian Province (China) and in Baguio district (Philippines), showed that the number of mineral deposits decreases as distance to geological controlling factors increases. This observation can be modeled using a multifractal singularity model, which can be expressed as a power-law relation between the density of mineral deposits (*ρ*) and the buffer width (*ε*) of geological features. Data sets for *ρ* and *ε* plotted on a log–log graph can be fitted by a straight line. The singularity index is equal to 2+ the gradient of the fitted line, and can provide a measure of the controlling function of geological factors on mineralization. Furthermore, this measure can represent the weights of geological controlling features in mineral prospectivity mapping. The advantage of the proposed method is that it can be easily implemented with GIS support, and can provide an objective measure of the controlling function of geological features on mineralization. A potential application of this approach is quantification of the spatial relationships between geological features and geological hazards, such as earthquakes and landslides.

## Method

A singular physical process refers to anomalous amounts of energy release or material accumulation within a narrow spatio–temporal interval^10^. Based on multifractal theory, Cheng^10^ proposed the singularity mapping method, which is now increasingly applied in GIS-based mineral exploration and geoscience data processing^11^,^12^. In the context of fractal and multifractal theory, the number of mineral deposits *N*(*ε*) of a *D*-dimensional set versus the scale of *ε* can be expressed as follows:^13^,^14^,^15^,^16^,^17^,^18^,^19^


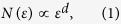


where, *d* is the fractal dimension, and ∝ means proportional to. Furthermore, the density of mineral deposits *(ρ)* versus *ε* can be expressed as:


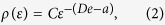


where *ε* denotes the buffer width around geological controlling features. *D*e is Euclidean dimension of space, and *a* is singularity index, and *C* is a constant. In this case, *D*e = 2. The expression can be written as:


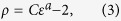


When *a* < 2, there is a significant spatial correlation between specific geological features and mineral deposits, with lower *a* (<2) values indicating an increasingly significant spatial correlation. A value of *a* > 2 suggests a negative spatial association, meaning that the number of mineral deposits increases as distance to geological features increases. The case of *a* = 2 suggests spatial independence, meaning that the spatial distribution of mineral deposits is not spatially related to geological features.

The following are the steps for calculating the singularity index *a*:

1. Select a geological feature (e.g., fault) that is related to the studied mineralization;
2. Supported by GIS, implement buffer analysis using *i* number of rings with an interval of *k* km (e.g., 0.5 km), and obtain a data set for buffer width (*ε*_*i*_);
3. Count the cumulative number of mineral deposits (*N*_i_) that fall into the geological feature buffer zone with different buffer widths (*ε*_*i*_);
4. Calculate the density of mineral deposits (*ρ*_*i*_) using *N*_i_/*ε*_*i*_;
5. Draw a log–log plot of *ρ*_*i*_ versus *ε*_*i*_, to which a straight line can be fitted. The gradient of the fitted line is *a*–2.

**Figure 4:**
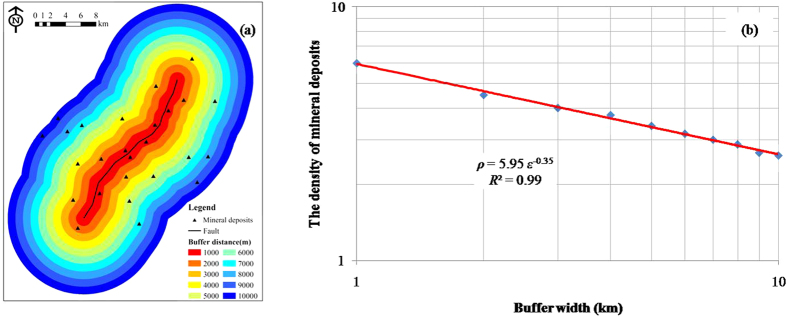
(**a**) Artificial example illustrating the singularity index calculation method; (**b**) a Log–log plot of density of mineral deposits versus buffer width of faults.

Taking a single fault as an example, 10-ring buffer analysis with an interval of 1 km was carried out (Fig. 4a). The number of mineral deposits occurring within each buffer zone was counted. The resulting data sets for the buffer width and the density of mineral deposits were plotted on a log–log graph, from which a fitted straight line could then be obtained, giving *ρ* = 5.95 *ε*^−0.35^(Fig. 4b). In this case, *a* = 1.65, i.e. <2, therefore suggesting a significant spatial correlation between the fault and the mineral deposits.

## Additional Information

**How to cite this article**: Zuo, R. A nonlinear controlling function of geological features on magmatic–hydrothermal mineralization. *Sci. Rep.* **6**, 27127; doi: 10.1038/srep27127 (2016).

## Figures and Tables

**Table 1:** Singularity index *a* versus student’ *t* value for geological features.

Case	Geological features	Singularity index *a*	Student’s *t*
Skarn Fe deposits in Fujian Province, China	Jurassic to Cretaceous intrusions	1.35	2.26
	C-P formation	1.30	4.63
	NNE–NE-trending faults	1.58	2.14
Epithemal Au mineralization in Baguio district, Philippines	NE-trending faults/fractures	1.58	2.84^8^
	NW-trending faults/fractures	1.92	−0.05^8^
	Batholithic pluton margins	1.59	2.88^8^
	Porphyry pluton contacts	1.54	3.10^8^
